# Optimizing imatinib sampling strategies through a population approach: a crucial step to model-informed precision dosing validation for point-of-care therapeutic drug monitoring

**DOI:** 10.1186/s40360-026-01181-5

**Published:** 2026-07-07

**Authors:** Yuan J. Pétermann, Junrui Chen, Lavinia Alberi, Simon Alexandra, François Veuve, Eva Choong, Yann Thoma, Roberto Rigamonti, Bruno Da Rocha Carvalho, Sandro Carrara, François R. Girardin, Thierry Buclin, Chantal Csajka, Monia Guidi

**Affiliations:** 1 Centre for Research and Innovation in Clinical Pharmaceutical Sciences, Lausanne University Hospital, University of Lausanne, Rue du Bugnon 17, Lausanne, CH-1011 Switzerland; 2 Department of Clinical Pharmacology, Lausanne University Hospital and University of Lausanne, Lausanne, Switzerland; 3 Bio/CMOS Interfaces Lab, École Polytechnique Fédérale de Lausanne, Rue de la Maladiere 71b, Neuchâtel, 2000 Switzerland; 4 VitalizeDx, Sarl, Lausanne, 1066 Switzerland; 5 VitalizeDx Eu Srl, 34121 Trieste, Italy; 6 School of Engineering and Management Vaud, HES-SO University of Applied Sciences and Arts Western Switzerland, Yverdon-les-Bains, 1401 Switzerland; 7 School of Pharmaceutical Sciences, University of Geneva, University of Lausanne, Geneva & Lausanne, Switzerland; 8 Institute of Pharmaceutical Sciences of Western Switzerland, University of Geneva, University of Lausanne, Geneva & Lausanne, Switzerland

**Keywords:** Therapeutic drug monitoring, Model-informed precision dosing, Imatinib, Optimal sampling strategy, Point-of-care testing

## Abstract

**Background:**

Imatinib therapeutic drug monitoring (TDM) contributes at optimizing exposure, yet its implementation is limited by logistical constraints, including strict sampling time requirements. Model-Informed Precision Dosing (MIPD) offers a promising approach to dosage individualization by leveraging population pharmacokinetic (popPK) models, thereby mitigating the constraints of sample collection time. Integrating MIPD and Point-of-Care (POC) analytical methods in ambulatory settings could further improve TDM feasibility and accessibility. This study serves as a proof of concept for MIPD integration in the workflow of imatinib TDM. To identify optimal sampling time for predicting imatinib steady-state trough concentrations (C_min, SS_) using a popPK model for an MIPD application.

**Methods:**

A popPK model developed from data of 146 patients (244 concentrations) was used to simulate individual concentration-time profiles of 1000 patients under standard dosing (400 mg once or twice daily) from treatment initiation to steady-state (reached after 11 days of treatment). Empirical Bayes estimates were generated from single or paired sampling time points and used to predict C_min, SS_. Predictive performance was evaluated using mean prediction error, coefficient of determination, root mean square prediction error (RMSPE), and successful prediction rates based on recommended therapeutic ranges.

**Results:**

Sampling 5–24 h and 1–12 h post-dose for once and twice daily administrations, respectively, yielded an RMSPE < 41% and a successful prediction rate around 70%.

**Conclusion:**

The proposed time windows provide flexible sampling strategies, confirming the benefits of MIPD for the convenience of imatinib TDM, thus improving the clinical feasibility of POC TDM.

**Supplementary Information:**

The online version contains supplementary material available at https://doi.org/10.1186/s40360-026-01181-5.

## Introduction

In the evolving landscape of precision medicine, the advancement towards precision medicine aims to tailor drug dosing to individual patient characteristics, optimising therapeutic outcomes, while simultaneously minimising adverse effects [1, 2]. One of the key strategies enabling this approach is Therapeutic Drug Monitoring (TDM), which involves the monitoring of drug concentrations in biological fluids at appropriately chosen sampling times. By evaluating whether individual drug exposure is subtherapeutic, excessive, or within the targeted range, TDM supports informed dosage adjustments to achieve optimal pharmacodynamic (PD) outcomes, when clear pharmacokinetic (PK) - PD relationships have been established. Since its early beginning in the 1920s, TDM has been recommended for a wide range of therapeutic agents across numerous medical disciplines, including oncology, infectious diseases, psychiatry, and immunology, encompassing anticancer drugs, biologics, antiretrovirals, anti-infectives, and psychotropics [1]. Despite its clinical value, TDM presents several limitations that must be addressed to realize its full potential in clinical practice. Logistical and economic factors, such as limited access to analytical laboratories and the necessary infrastructure for timely drug concentration measurements, can hinder the routine implementation of TDM [1, 3]. The process is inherently laborious, complex, and time-consuming, involving extensive pre-analytical, analytical, and post-analytical work from many actors (nurses, specialised laboratory staff, medical doctors), making it prone to errors at multiple levels [2, 4]. To allow dosage adjustments, classical TDM typically requires drawing blood samples at trough time once the pharmacokinetic steady-state is achieved [5]. This represents a further significant limitation, as inaccurate sampling times compromise the reliability of interpretation, potentially delaying therapeutic interventions and reducing the possibility of adapting treatments to rapidly evolving clinical conditions [5].

A truly transformative approach is the integration of Model-Informed Precision Dosing (MIPD) and Point-of-Care (POC) testing into TDM, which together have the potential to revolutionise drug individualisation [5–7]. Unlike conventional laboratory-based TDM, which relies on centralized infrastructure and involves complex, time-consuming sample transport and pre-analytical processing, POC testing refers to rapid, decentralized analytical methods performed directly at or near the site of patient care [6, 8]. When coupled with a POC, this strategy gains practical value by enabling rapid data collection, fast drug level measurements, and dosage adjustment directly at the patient’s bedside [6–8]. This integration reduces logistical delays and minimizes the number of intermediary steps between sample collection and clinical decision-making, thereby improving responsiveness and reducing the potential for process error [6–8]. By leveraging previously developed population pharmacokinetic (popPK) models and Bayesian forecasting methods, MIPD enables accurate prediction of individual PK profiles and resulting drug exposures, even based on a single or a few concentration measurements, not necessarily taken at trough time [9]. This approach eliminates the necessity of waiting for steady-state conditions, allowing earlier and more flexible drug monitoring that is better aligned with the patient’s evolving clinical status [5, 6]. POC testing complements this approach by providing rapid, decentralized analytical methods performed directly at or near the site of patient care enabling timely data collection and fast drug level measurements [6–8]. The integration of MIPD and POC within TDM allows for dosage adjustment directly at the patient’s bedside, reducing logistical delays and minimizing the number of intermediary steps between sample collection and clinical decision-making, compared to conventional laboratory-based TDM. As a result, this combined strategy improves responsiveness and reduces the potential for process error [5, 6, 10]. Importantly, the predictive accuracy and clinical utility of MIPD tools depend critically on the quality and relevance of the underlying popPK model. The implemented model must adequately represent the target population, accounting for its demographic, physiological, and clinical characteristics [7]. Therefore, to optimize reliable individual predictions, the model should ideally be developed and validated in a population comparable to, or directly representative of, the intended population of use.

Given its long-term oral administration in an outpatient setting, imatinib represents a particularly relevant model-case to demonstrate the implementation of MIPD combined with POC-based TDM in oncology [11]. As a first-generation tyrosine kinase inhibitor, imatinib exhibits marked inter-individual PK variability, making standardized dosing suboptimal for many patients [12, 13]. Imatinib exhibits linear PK and has a prolonged terminal half-life of approximately 18 h [12, 14]. Clear exposure-response relationships have been documented, with traditional efficacy targets defined by steady-state trough concentrations above 1000 ng/mL for chronic myeloid leukemia (CML) and above 1100 ng/mL for gastrointestinal stromal tumours (GIST), while concentrations exceeding 2000–3000 ng/mL are associated with an increased risk of toxicity [10, 12, 15]. Although trough concentrations are traditionally used in routine TDM, recent studies suggest that peak concentrations might be related to adverse events such as fluid retention, bone pain or fatigue [16, 17]. Yet, these relationships are for now not well established, and trough concentrations remains the primary TDM metric with well-defined therapeutic boundaries in consensus guidelines. Moreover, TDM-guided dosing of imatinib has been associated with improved clinical outcomes over patients deprived of TDM assistance, including higher rates of major molecular and cytogenetic responses at 12 months, with 63% and 37% remission rates, respectively [12, 13, 18]. A recent cost-effectiveness analysis also demonstrated that TDM yields both improved quality-adjusted life years and lower overall costs compared to fixed dosing strategies [12, 19, 20]. As of 2021, the International Association of Therapeutic Drug Monitoring and Clinical Toxicology (IATDM-CT) officially recommends imatinib TDM for patients with CML and GIST [15]. While POC approaches for imatinib have been explored previously, they have not yet been combined with MIPD, to our knowledge [8, 10]. On the other hand, an integrated approach for TDM allows for real-time data collection and dosage adjustment at the point of care, such as in ambulatory medical facilities [11].

A key practical constraint remains, which is the number and timing of samples required to accurately inform model-based predictions [21]. In addition to the appropriateness of the underlying model, the predictive performance of MIPD depends strongly on the informativeness of the sampling schedule, which must be carefully designed to balance practicality with accuracy, ensuring sufficient accuracy and clinically actionable dosing recommendations [21]. This study aimed to evaluate the predictive accuracy of various sampling times for forecasting the steady-state trough concentration of imatinib. It provides a key step toward validating the clinical feasibility of MIPD coupled to a POC TDM strategy, using imatinib as a case study. It follows on from the development of a POC analytical method based on memristive biosensors capable of quickly delivering accurate drug concentration measurements [8].

## Method

### Studied population and data

Ambulatory and hospitalized adult patients receiving imatinib and undergoing routine TDM at the University Hospital of Lausanne between 2012 and 2024 were retrospectively included in this study. At each sampling time point and when available, concomitant medications, demographic characteristics (i.e., age, body weight, height, sex), and relevant clinical parameters such as albumin or alpha-acid glycoprotein levels were collected on the sampling time. All enrolled patients were assumed to be at steady state, as per TDM practice at our hospital. Records with missing or unreliable information regarding the timing of drug intake and/or sampling were excluded. Imatinib concentrations were assessed by high-performance liquid chromatography coupled with tandem mass spectrometry, with a lower limit of quantification (LLOQ) of 20 ng/mL [22].

### Population pharmacokinetic analysis

PopPK analyses and simulations were conducted using MonolixSuite™ (version 2024R1) [23]. Statistical analyses, data management, and graphical exploration were performed in R (version 4.4.2). Two concentration measurements below the LLOQ were excluded from the analysis.

### Base and covariate models

Model selection followed a classical stepwise procedure, comparing structural models with increasing numbers of compartments, under the assumption of linear absorption and elimination. PK parameters were assumed to follow a log-normal distribution and, given the oral administration of imatinib, represent apparent values. As limited data were available during the absorption phase, the first-order absorption rate constant (ka) was fixed to a value close to those reported in published popPK models [14, 24, 25], providing a time to maximum concentration (T_max_) consistent with the literature range of 1.5–4.0 h [26, 27]. Inter-individual variability (IIV) was assessed sequentially for each parameter, except for ka, and incorporated into the model when it significantly improved the fit. Various error structures (additive, proportional, and mixed) were evaluated to best account for residual unexplained variability (RUV).

Covariate analyses considered relevant clinical and demographic factors previously associated with imatinib PK [24]. Variables with more than 5% missing data were not considered in the covariate analyses. Concomitant medications known to influence imatinib metabolism, such as cytochrome P450 3A4 (CYP3A4) inhibitors, were not documented in the present dataset and could not be incorporated into the analysis [28]. Since the intended application of the model was integration in MIPD, model development prioritized parsimony to facilitate integration into point-of-care workflows. Consequently, covariates were retained in the final model only if they met statistical criteria and demonstrated a clinically relevant effect magnitude (defined as > 20% change in parameter value), relying on *a posteriori* Bayesian forecasting to compensate for minor phenotypic variability.

### Model selection and evaluation

Comparisons between nested model structures were based on differences in the objective function values (ΔOFV), which approximately follow a chi-square distribution. During the forward selection process for both the base model development and the forward covariate analysis step, a significance level of 0.05 (e.g., ΔOFV < -3.84 for one additional estimated parameter) was applied. In the backward selection step, all covariates previously included were re-evaluated by sequentially removing them from the full model, and a stricter threshold of 0.01 (ΔOFV > 6.63 for one parameter removal) was used to determine which covariates to retain in the final model. Besides statistical criteria, model development was further guided by diagnostic tools, including goodness-of-fit plots, relative standard errors (RSE) of parameter estimates, and amplitude of the identified covariate effect as well.

Internal validation of the final imatinib popPK model was performed using a non-parametric bootstrap method based on 2000 datasets resampled from the original cohort. This has allowed comparison of the final model parameter estimates with their bootstrap estimated medians and percentile-based 95% confidence intervals (CI_95%_). Additionally, the model’s predictive performance was assessed through prediction-corrected visual predictive checks (pcVPC) generated from 1000 simulations. The resulting 90% confidence intervals were compared to the observed concentration-time 5th, 50th, and 95th percentiles.

### Optimal sampling strategy

To identify the optimal sampling strategy, an exhaustive all-combination approach was applied for both single-sample and two-sample designs [21]. A virtual cohort of 1000 patients receiving the most common dosing regimens observed in the studied population (400 mg once or twice daily) was generated through Monte-Carlo simulations using the final popPK model. To ensure clinically relevant pharmacokinetic profiles, unreliable simulated patients were excluded by selecting a population whose half-lives (t_1/2_) distribution was centred around the observed median value and encompassed the range observed in the studied population. Concentration–time profiles were simulated at hourly intervals from day 1 to day 11, corresponding to a duration ensuring steady-state in all individuals. A random RUV component was assigned to all simulated values within each individual profile. The trough concentration at steady-state (i.e., 264 h after treatment initiation), devoid of RUV, was defined as the true C_min, True_.

The single sample strategy considered a single simulated concentration at each possible sampling hour (1–264 h after treatment initiation). For the 400 mg once-daily regimen, a two-sample strategy was evaluated by assessing all possible combinations within two predefined windows: 1–48 h (early phase) and 240–264 h (steady-state). The extension of this multi-sample analysis to the 400 mg twice-daily regimen was defined as conditional to be performed only if the two-sample strategy demonstrated significantly superior predictive performance compared to the single-sample approach in the once-daily group. In each case, empirical Bayes estimates (EBEs) were derived using the final popPK model in MonolixSuite™ and the simulated concentrations at the respective sampling times. For all tested strategies, the EBEs were used to predict the trough concentration at steady-state in the simulated individual (C_min, Pred_). C_min, Pred_ values were then compared to the C_min, True_ using mean relative prediction error (MPE [%], Eq. (2)), root mean square relative prediction error (RMSPE [%], Eq. (3)), and a categorical success metric (Successful Prediction Rate [%]). The latter was defined as the proportion of cases in which both C_min, Pred_ and C_min, True_ values fell within the same range, i.e. both underdosed (below 1100 ng/mL), correctly dosed (between 1100 and 2000 ng/mL), and overdosed (above 2000 ng/mL).

1 $$\:{PE}_{i}=\mathrm{l}\mathrm{n}\left({{C}_{min,\:\:\:Pred}}_{i}\right)-\mathrm{l}\mathrm{n}\left({{True\:C}_{min,\:\:\:True\:}}_{i}\right)$$

2 $$\:{\mathrm{MPE}}\:\left( \% \right) = \left( {{\mathrm{exp}}\left( {\frac{1}{{\mathrm{n}}}\sum\limits_{i = 1}^n {P{E_i}} } \right) - 1} \right){\mathrm{*}}100$$

3 $$\:{\mathrm{RMSPE}}\:\left( \% \right) = \left( {{\mathrm{exp}}\left( {\sqrt {\frac{1}{{\mathrm{n}}}\sum\limits_{i = 1}^n {P{E_i}^2} } } \right) - 1} \right){\mathrm{*}}100$$

An absolute MPE ≤ 30% was considered acceptable, while values ≤ 15% were defined as low, consistent with previously proposed methodology [21]. Regarding RMSPE, thresholds were scaled to the RUV defining values inferior to 1.2 × RUV as good, 1.2–1.5 × RUV as acceptable, and superior to 1.5 × RUV as unacceptable. Sampling strategies meeting these thresholds, when combined with a high successful prediction rate (> 80%), were retained as clinically relevant.

## Results

### Studied population

A total of 534 observed plasma concentrations of imatinib from 263 patients were initially available. Following the exclusion of 290 observations with unreliable or missing information, 244 samples from 146 patients were retained for the popPK analysis. Table 1 summarizes the dosing regimens. Age and sex were available for all patients, with 92 males (63%) and 54 females (37%), and a median (range) age of 62 (18–90) years. For all other covariates considered, more than 95% of the data were missing due to the outpatient nature of the population and were thus discarded from the covariates analysis. The median (range) number of samples per patient was 1 [1–13], and the median time after last dose intake was 17 h (range: 0.5–29 h). Only a small proportion of samples (8%) were collected prior to 3 h.

**Table 1:** Dosing schemes in the studied population

Dosing [mg]	Intake per day	*n* (%)
100	1	4 (3)
200	1	8 (5)
200	2	3 (2)
300	1	8 (5)
400	1	106 (73)
400	2	8 (5)
600	1	6 (4)
800	1	3 (2)

### Population pharmacokinetic model

A one-compartment model with first-order absorption and linear elimination best fitted the data consistently with literature [24]. Inclusion of IIV on clearance (CL) resulted in a significant improvement in model fit (ΔOFV = -60, *p* < 0.001), which was further improved with the addition of IIV on the volume of distribution (V_d_, ΔOFV = -8.3, *p* < 0.01). As explained, ka had to be fixed to 1.28 h⁻¹ due to limited data in the absorption phase, yielding a model-predicted T_max_ of approximately 3 h, concordant with the literature [26, 27]. Sensitivity analyses evaluating alternative ka demonstrated that fixing ka to 1.28 h^− 1^ exerted negligible influence on the estimation of model parameters. A proportional error model was not significantly different from a mixed error model and was retained for RUV (ΔOFV = -0.00, *p* > 0.1).

Although previous studies have identified age as a significant covariate, only sex was found to be significant in our analysis (ΔOFV = -6.9, *p* < 0.01), with 15% decrease in CL in females compared to males, consistent with prior reports [14, 24]. Given the relatively low magnitude of effect and the absence of inter-individual variability reduction, the covariate was not retained and the structural model was considered as the final one.

**Table 2:** Final population apparent PK parameter estimates of imatinib and bootstrap results

Parameter		Final model	Bootstrap (*n* = 2000)
		Estimate (RSE%)	Median (CI_95%_)
*CL [L/h]*		11 (4.5)	11 (9.9–12)
	*ω*_*Cl*_ *[CV%]*	33 (14)	29 (18–19)
*V*_*d*_ *[L]*		365 (11)	394 (285–538)
	*ω*_*Vd*_ *[CV%]*	58 (19)	74 (40–100)
*ka [h* ^*− 1*^ *]*		1.28 FIX	
*σ*_*prop*_ *[CV%]*		34 (6.6)	34 (29–39)

**ka**: typical first-order absorption rate constant; **V**_**d**_: typical central volume of distribution; **CL**: typical clearance; **ω**: inter-individual variability parameter, **σ**_**prop**_: proportional residual error, **CI**_**95%**_: percentile-based confidence interval between 2.5% and 97.5%; **RSE**: relative standard error. **CV** (%) for **ω** and **σ**_**prop**_ is calculated as follows:$\:CV=\:\sqrt{({e}^{{\omega\:}^{2}}-1)}$

**Fig. 1:**
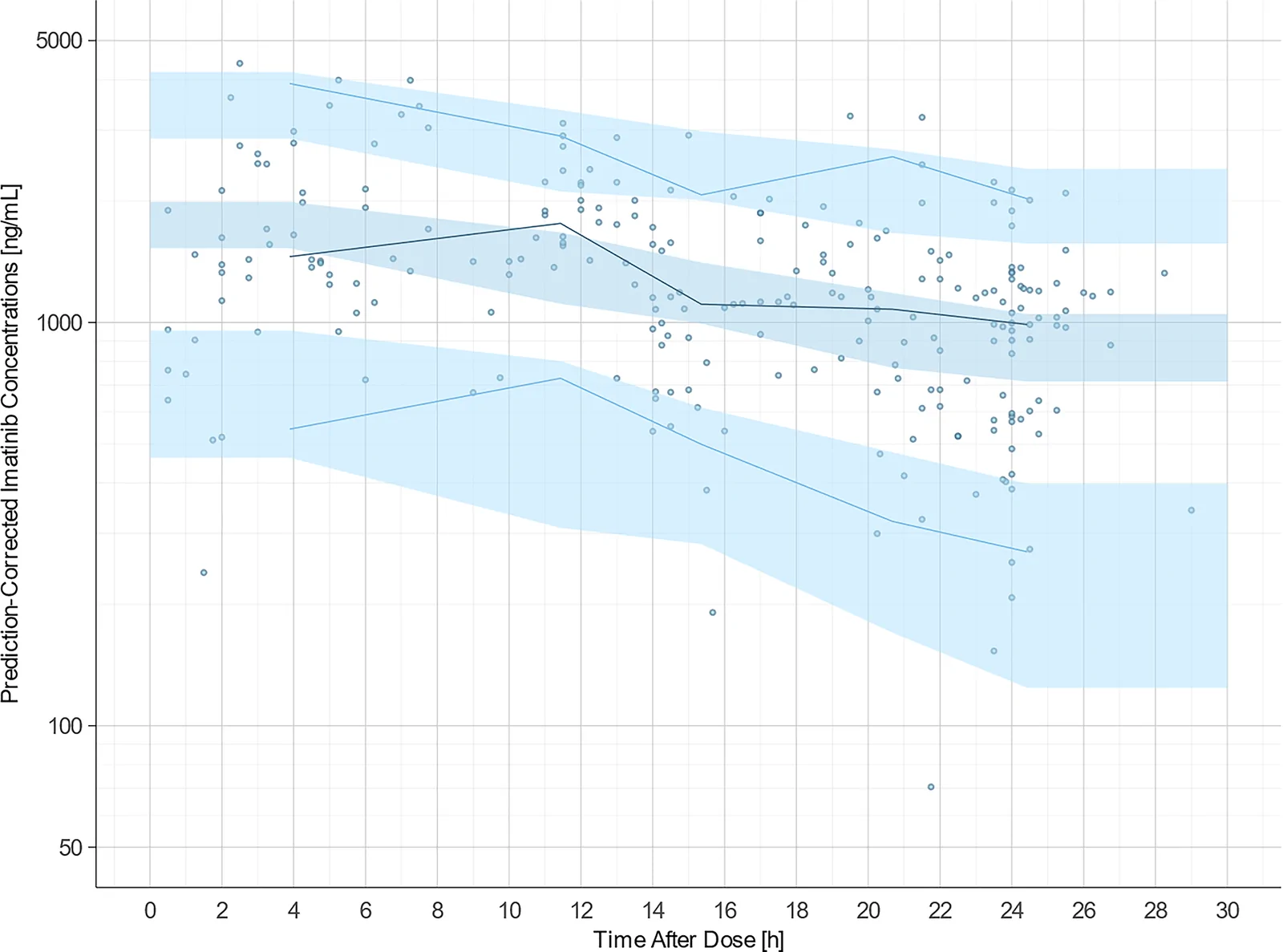
Imatinib prediction-corrected visual predictive check (pcVPC) of the final model in log-transformed y axis. The points represent the prediction-corrected observed concentrations, the solid lines represent the 5th, 50th (median), and 95th percentiles of the observed concentrations. Shaded areas depict the 90% prediction intervals around the 5th, 50th (median), and 95th model-predicted percentiles

Table 2 shows the final parameter results with their bootstrap evaluations. Prediction-corrected visual predictive checks (pcVPC), presented in Fig. 1, along with goodness-of-fit (GOF) plots (Supplementary Figure S1) and bootstrap results, confirmed the adequacy and robustness of the final model.

### Optimal sampling strategy

**Fig. 2:**
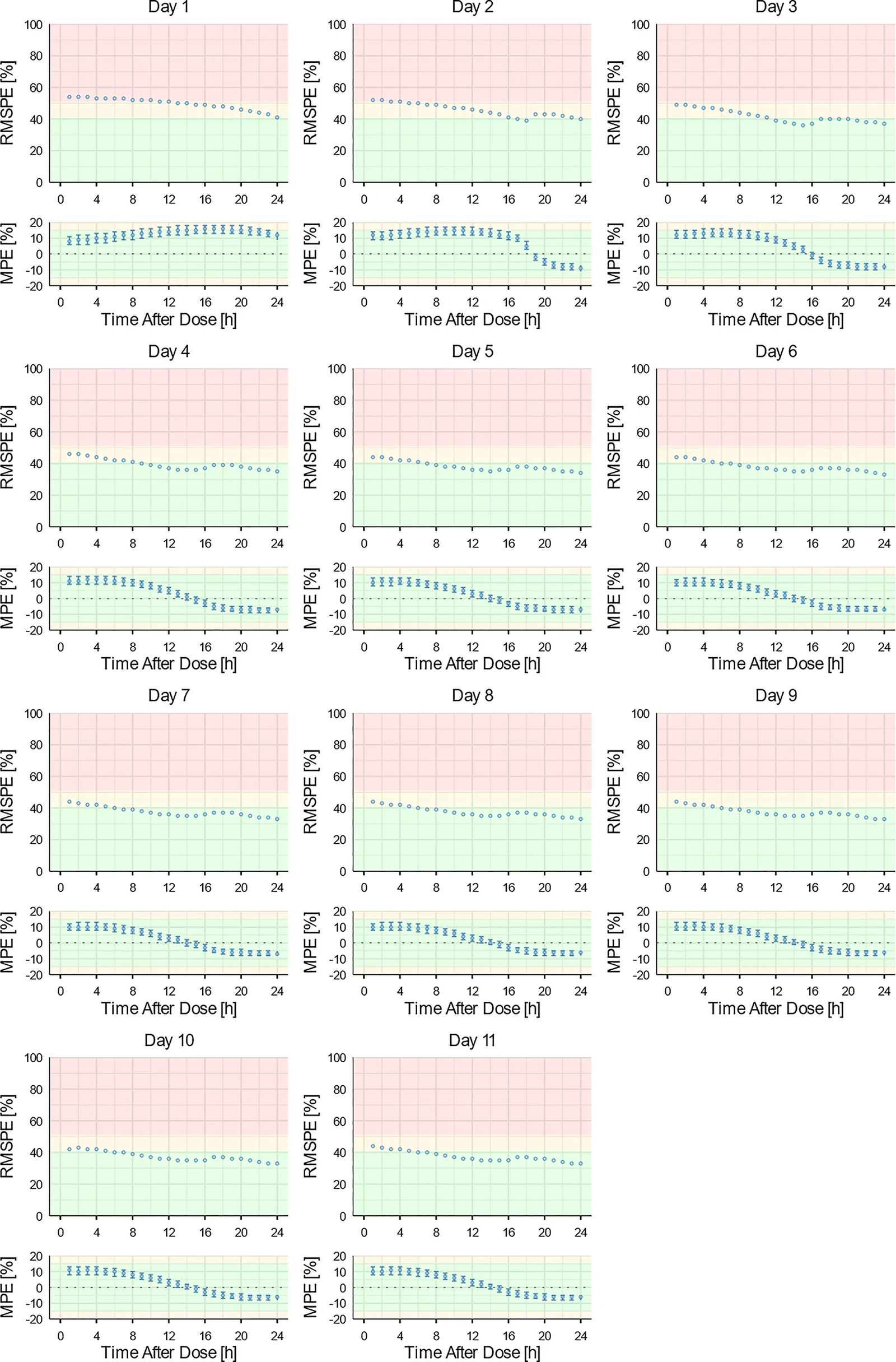
Predicted steady-state trough concentration (C_min, Pred_ at 24 h post dose on day 11) error metrics from the final popPK model using a single sampling time between day 1 and day 11, for 1000 simulated patients at 400 mg once daily. Blue points denote each sampling scenario (X axis) and the corresponding Mean Predictive Error (MPE [%]) and Root Mean Square Prediction Error (RMSPE [%]). Shaded regions indicate performance tiers: green for good (RMSPE ≤ 41% or |MPE| ≤ 15%), yellow for acceptable (41% < RMSPE ≤ 51% or 15% < |MPE| ≤ 30%), and red for unacceptable (RMSPE > 51% or |MPE| > 30%). Error bars show the 95% confidence intervals for MPE

**Fig. 3:**
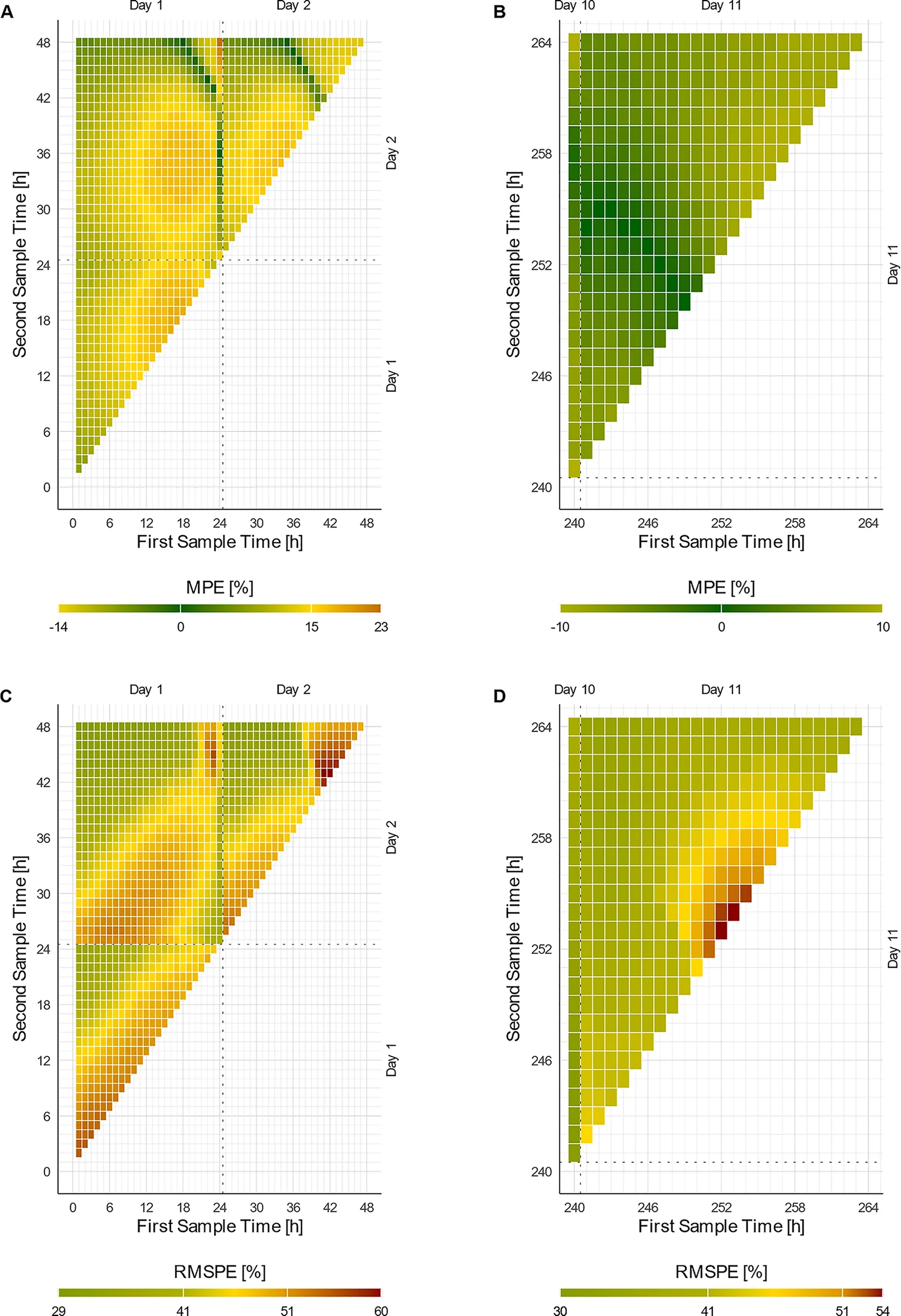
Heat maps of predictive performance of the steady-state trough concentrations (C_min, True_) at 24 h post dose on day 11 across paired sampling times since the first dose on days 1–2 (left panels) and days 10–11 of treatment (right panels), for 1000 simulated patients at 400 mg once daily. Panels A & B show Mean Predictive Error (MPE): green cells indicate low bias (between − 15%< MPE ≤ 15%) and yellow cells acceptable bias (15% < |MPE| ≤ 30%). Panels C & D show Root Mean Square Prediction Error (RMSPE): green cells denote good precision (RMSPE ≤ 41%), yellow cells acceptable precision (41% < RMSPE ≤ 51%), and red cells unacceptable precision (RMSPE > 51%)

For the 400 mg once-daily regimen, the estimation bias of all sampling strategies was low to moderate, with MPE ranging from − 9% to 16% and − 14% to 23% for the single- and two-sample strategies, respectively (Figs. 2 and 3; Supplementary Table S2).

**Fig. 4:**
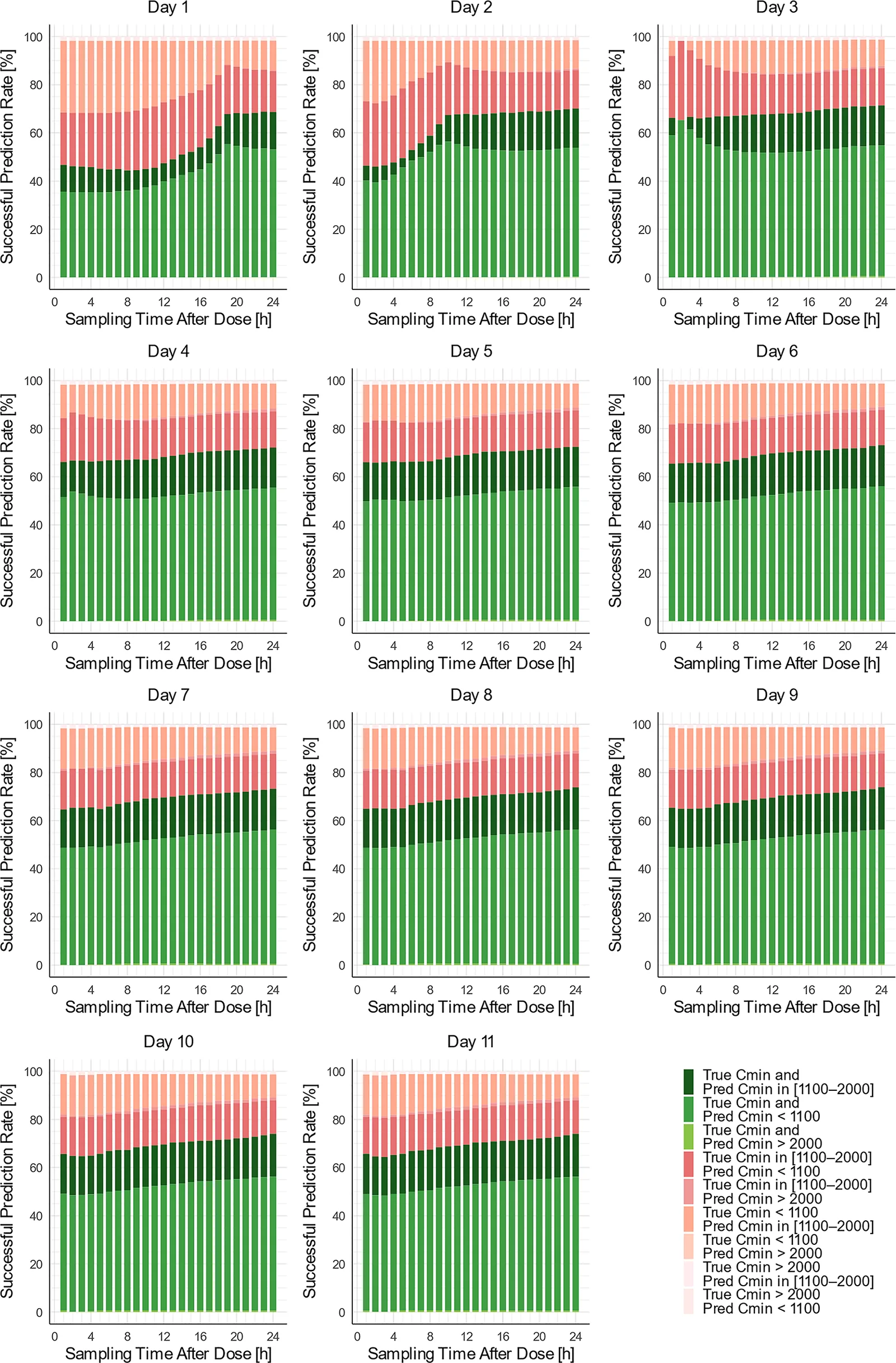
Proportion of virtual individuals (n_tot_ = 1000) under 400 mg once daily for which the predicted (C_min, Pred_) and true (C_min, True_) trough concentrations (i.e., 24 h post dose on day 11–264 h) fell within the same range (< 1100, 1100–2000, > 2000 ng/mL). Concordant results in green shades, discordant in yellow to red shades, based on the therapeutic range given in [15]

Prediction accuracy varied more widely, with RMSPE values of 33–54% and 29–60% for the single- and two-sample strategies, respectively. Successful prediction rates ranged from 44% to 74% and 44% to 75% for the single- and two-sample strategies, respectively (Fig. 4; Table 3). Overall, the two-sample strategy yielded results comparable to the single-sample approach, offering no improvement in predictive performance.

**Table 3:** Proposal of single sampling times

	Day after treatment initiation [d]	Proposed sampling times of the day [h]	MPE Range [%]	RMSPE Range [%]	Successful prediction rate range [%]
400 mg QD	1	24	12	41	69
	2	16 to 18, 23 to 24	-9 to 11	39 to 41	68 to 70
	3	11 to 24	-8 to 10	36 to 41	68 to 71
	4	8 to 24	-7 to 10	35 to 41	67 to 72
	5	6 to 24	-7 to 10	34 to 41	66 to 72
	6	5 to 24	-7 to 10	33 to 41	66 to 73
	7	5 to 24	-7 to 10	33 to 41	65 to 73
	8	5 to 24	-7 to 10	33 to 41	65 to 74
	9	5 to 24	-6 to 10	33 to 41	65 to 74
	10	5 to 24	-6 to 10	33 to 41	66 to 74
	11	5 to 24	-6 to 10	33 to 41	66 to 74
400 mg BID	1	6 to 24	7 to 10	37 to 41	73
	2	1 to 24	5 to 9	32 to 39	73
	3	1 to 24	-2 to 9	30 to 36	73 to 78
	4	1 to 24	-2 to 7	30 to 34	73 to 78
	5	1 to 24	-2 to 6	29 to 33	74 to 78
	6	1 to 24	-2 to 5	29 to 32	75 to 78
	7	1 to 24	-2 to 5	29 to 32	75 to 77
	8	1 to 24	-2 to 5	29 to 32	76 to 77
	9	1 to 24	-2 to 5	29 to 32	76 to 78
	10	1 to 24	-2 to 5	29 to 32	76 to 78
	11	1 to 24	-2 to 5	29 to 32	76 to 78

**QD**: once daily; **BID**: twice daily; **MPE**: Mean Predictive Error; **RMSPE**: Root Mean Square Precision Error; **Successful Prediction Rate**: Percentage of steady-state C_min_ values for which the predicted and true concentrations fell in the same category (< 1100, 1100–2000, > 2000 ng/mL). Only sampling strategies with good RMSPE (≤ 41%) were selected

For the 400 mg twice-daily regimen, MPE ranged from − 2% to 10% for the single-sample strategy, again indicating low to moderate bias (Figure S3.1; Supplementary Table S2), while RMSPE values ranged from 29% to 43%. Successful prediction rates ranged from 73% to 78%.

Based on the estimated RUV, sampling times were defined as ‘good’ if they yielded an RMSPE ≤ 41% and an absolute bias (|MPE|) ≤ 15%. Performance was considered ‘acceptable’ if RMSPE fell between 41% and 51% and if |MPE| was between 15% and 30%. Any values exceeding these limits were classified as ‘unacceptable’. Table 3 summarizes the proposed sampling time for 1-sample strategies.

## Discussion

This study critically addressed a practical barrier in integrating MIPD with a POC TDM system: the identification of optimal and acceptable sampling strategies. By systematically evaluating the timing and number of samples required to predict steady-state imatinib trough concentrations accurately, we aimed to establish a pragmatic new framework that balances predictive accuracy with clinical feasibility. The results provide a proof-of-concept, which confirms that the sampling design is a key-decisive determinant of predictive performance, thereby supporting the development of efficient and clinically actionable MIPD–POC workflows.Although several imatinib models have been published, they did not apply to the studied population due to the limited availability of identified relevant covariates, rendering direct application or external validation of published models infeasible [14, 24, 25, 29]. Consequently, a simplified yet purpose-fit model was developed specifically for MIPD using Bayesian forecasting, which demonstrates robust predictive performances. The selection of a one-compartment structural framework with first-order linear elimination aligns with the standard methodology utilized in the majority of published imatinib population pharmacokinetic models [24]. Furthermore, the final parameter estimates fall well within established ranges found in a recent systematic and meta-analysis, confirming the physiological plausibility of the model [24]. Specifically, the apparent clearance of 11 L/h sits within the typical literature range of 7.3 to 17.3 L/h, while the volume of distribution of 365 L aligns with reported values spanning from 128 to 430 L. Additionally, the fixed absorption rate constant of 1.28 h⁻¹ is consistent with literature estimates ranging from 0.329 to 2.06 h⁻¹. Although the baseline dataset is relatively modest and predominantly composed of steady-state trough samples, which inherently limits the dense mathematical characterization of the early absorption and distribution phases, the model’s fundamental robustness is supported by the close alignment of its parameter estimates and derived half-life with other models found in the literature [24]. Despite this constraint, the model yielded derived parameters, i.e. half-life and T_max_ consistent with those reported in the literature [14, 24, 25, 29–31]. Therefore, although the baseline dataset was relatively modest and predominantly composed of steady-state trough samples, which inherently limited the characterization of the early absorption and distribution phases, the close alignment of model’s estimated and derived parameters with those found in the literature supports its reliability and plausibility [24]. Finally, while some historical models identified age as a significant covariate for clearance, this effect was not observed in our cohort [24]. This is primarily because our older, adult-focused demographic distribution (median age: 62 years; 25th percentile: 49 years) lacked the broader age range and sample size per age stratum required to fully capture such an effect. Acceptable and good prediction of steady-state trough concentrations depends critically on both the timing and number of samples. Given the consistently low bias across time points, RMSPE was the principal metric driving the predictive value of sampling strategies, in line with the methodology proposed by Van der Meer et al. [21]. Our results demonstrate that predictive performance is not predominantly determined by the treatment day, but rather by the timing of sampling relative to the last dose intake.

For the single-sample strategy with 400 mg once daily dosing, RMSPE values dropped below the good upper threshold value (41%) mostly during the elimination and distribution phases. This pattern of acceptable precision of the trough concentration initiated on the first day and progressively expanded to the remaining days. As treatme.

nt approached steady-state, samples collected earlier than the trough became increasingly reliable. A similar trend was observed for the 400 mg twice daily regimen, where acceptable sampling windows were identified as early as the first day of treatment.

The results suggest that late post-dose samples are sufficiently informative to accurately estimate steady-state C_min_. The high successful prediction rates observed at these sampling times reflect a strong concordance between predicted C_min, Pred_ and true C_min, True_ values. This indicates that the risk of misclassifying patient exposure, whether underdosed, correctly dosed, or overdosed, remains low and clinically acceptable. While combining a day 1 sample with the appropriate one from day 2 dropped the RMSPE below the good threshold value (41%), these combinations did not offer a better performance than a single optimally timed sample at the trough of day 1. Consequently, the logistical burden of a second sampling point does not appear justified, as a single well-timed sample is sufficient to achieve appropriate predictive precision. Although simulation scenarios were restricted to the 400 mg once-daily and twice-daily regimens due to their high clinical prevalence in our cohort, other dosing regimens are expected to yield identical optimal sampling windows given the strictly linear PK profile of imatinib. Future clinical validation in independent patient cohorts will help confirm the generalizability of these flexible sampling strategies across a broader range of real-world dosages.

The distinct optimal sampling windows identified for the 400 mg once-daily and twice-daily regimens are primarily driven by the timing of the steady-state trough relative to the drug’s t_1/2_ of approximately 23 h. For the 400 mg once daily regimen, the trough occurs at 24 h post-dose, a point where plasma concentrations are predominantly determined by individual CL. Consequently, samples taken during the early distribution phase are more informative of the V_d_ than CL, leading to higher extrapolation errors. In contrast, the trough of the 400 mg twice a day regimen occurs at 12 h, a midpoint significantly influenced by the volume of distribution. Since early post-dose samples accurately inform V_d_, they provide a more robust basis for predicting the 12-hour trough before the clearance-dominant terminal phase begins. While the successful prediction rate provides a pragmatic summary of classification accuracy, this metric does not by itself guarantee optimal clinical decision-making. Classification within the same therapeutic category (i.e., under-, over-dosed, or correctly dosed) may obscure clinically meaningful deviations in absolute concentration values, particularly near threshold boundaries, which should still influence dosing decisions. Moreover, this interpretation depends on the choice of therapeutic targets, which may vary depending on the clinical condition of the patient [12, 15, 32]. These limitations underscore the risk of inappropriate interventions, even when categorical concordance is observed. Inaccurate predictions could lead to suboptimal dosage adjustments, potentially compromising efficacy or safety. Nonetheless, the consistently low MPE and RMSPE observed across the most informative sampling strategies provide reassurance regarding the accuracy of the C_min, Pred_ values. The convergence of multiple performance indicators (including a high successful prediction rate, a low bias, and a high precision) offers a robust body of evidence supporting the reliability and clinical applicability of the proposed sampling strategies for model-informed dosage individualization.

While this study establishes a theoretical framework for flexible sampling, these findings require prospective validation using the final POC device. Because the popPK model was developed using standard LC-MS/MS data, translation of those findings to another analytical method depends on strict analytical alignment. Future implementation must confirm that the memristive biosensor’s performance is either directly comparable to LC-MS/MS concentrations or cross-calibrated via a reliable conversion equivalent to guarantee predictive accuracy of our results.

A limitation of this study is the inherent circularity issue introduced by evaluating the flexible sampling windows on a virtual cohort simulated from our baseline population model. While this framework limits the formal assessment of robustness against structural model misspecification, the close structural alignment of our final parameters with standard consensus models suggests that the virtual population remains highly representative of typical clinical behaviour. Further external validation of the model and the proposed sampling strategy might tackle this limitation. An additional limitation relates to the fixation of the ka. Although, this assumption does not alter the modelling results, it could potentially influence the evaluated performance of early samples taken during the absorption. This limitation could shift these early hours outside the acceptable accuracy limits due to unaccounted inter-individual variability in absorption phase. Future studies involving dense, early-phase kinetic sampling are required to further investigate alternative absorption models and refine the boundaries of these early sampling windows.

The sampling strategies evaluated as suitable in this study are inherently dependent on the quality of the popPK model used to generate virtual patients’ representative of the population on which the MIPD would be used. Yet, observations suggest that the popPK model included in the MIPD, while important, does not need to perfectly represent the target population to remain clinically useful. Indeed, even when based on a suboptimal or poorly representative model, the a posteriori Bayesian approach leveraging observed concentrations can dynamically adjust initial predictions to properly predict patients’ exposure [33]. These findings rather underscore the critical importance of external validation, as emphasized in [21, 34, 35] to confirm the generalizability of the proposed sampling strategies.

## Conclusion

The study demonstrates that accurate prediction of imatinib trough concentrations at steady state is feasible with MIPD based on a single TDM sample, provided that sampling is performed at appropriate timepoints (typically 5–24 h and 1–12 h post-dose for 400 mg once and twice daily regimen, respectively). While the twice-daily regimen robustly met our clinical relevance thresholds (typically 1–12 h post-dose), the once-daily regimen proved more borderline (typically 5–24 h post-dose), peaking at a 74% successful prediction rate and failing to fully meet our predefined 80% target. These wider, non-trough sampling offers a highly practical and logistically feasible strategy that facilitates TDM optimization within routine, flexible outpatient workflows. Thus, this result constitutes a proof-of-concept for integrating MIPD with POC TDM for imatinib, and is encouraging for many other targeted oral anticancer agents and further critical treatments candidates to dosage individualization. Predictive performance is primarily determined by the timing of sample collection relative to the last dose, with sampling during the distribution or elimination phase yielding the most reliable results across both single- and two-sample strategies. These findings now warrant prospective validation using independent patient data, to confirm their applicability in clinical practice. Yet, the results of this study support flexible and pragmatic sampling schedules for MIPD and lay the groundwork for coupling it to appropriate POC analytical methods. As technology advances toward decentralized TDM, future clinical validation will be essential to assess the real-world impact of these strategies on treatment efficacy, safety, and patient outcomes.

## Supplementary Information

Below is the link to the electronic supplementary material.

Supplementary Material 1

Supplementary Material 2

## Data Availability

The data underlying this study contain sensitive participant information and are not publicly accessible. However, they can be obtained from the corresponding author (MG) upon reasonable request.
