# Supplementary material for: Optimizing imatinib sampling strategies through a population approach: a crucial step to model-informed precision dosing validation for point-of-care therapeutic drug monitoring
Source: BMC Pharmacol Toxicol. 2026 Jul 7;27:139. doi: 10.1186/s40360-026-01181-5 (PMC13632223; doi:10.1186/s40360-026-01181-5)
Supplement: Supplementary file 1 [file 40360_2026_1181_MOESM1_ESM.docx]

# 1. Supplemental Material

## Supplemental Material 1: Goodness-of-fit plots of the final population pharmacokinetic model of imatinib

**Figure S1**. Goodness-of-fit plots for imatinib population pharmacokinetic analysis. Panel A shows observed versus individual-predicted concentrations and Panel B observed versus population-predicted concentrations in log-transformed axis, with the line of identity (solid line) included for reference. Panels C and D depict individual weighted residuals (IWRES) plotted against individual predictions and time after dose, respectively, including the solid line at 0 indicating an ideal case deprived from prediction error. Panels E and F present normalized prediction distribution errors (NPDE) versus individual predictions and time after dose, and the solid horizontal line at zero represents the reference of unbiased predictions.

## Supplemental Material 2: Optimal sampling strategy results

Table S2 summarizes the MPE, RMSPE, and successful prediction rate for all sampling strategies evaluated for patients under imatinib at 400 mg once or twice daily dosing strategies. These include single-sample strategies tested hourly over 11 days and two-sample strategy for the 400 mg once daily regimen. Prediction success is defined as the proportion of cases in which the true and predicted trough concentrations fall within the same dosing category: underdosed (<1100 ng/mL), correctly dosed (1100–2000 ng/mL), or overdosed (>2000 ng/mL).

## Supplemental Material 3: Optimal sampling strategy results for 400 mg twice a day

**Figure S3.1.** Predicted steady-state trough concentration (C_min, Pred_ at 24 h post dose on day 11) error metrics from the final population PK model using a single sampling time between day 1 and day 11, for 1000 simulated patients at 400 mg twice daily. The dotted vertical lines at 12 h separate the 2 doses intakes within the day. Blue points denote each sampling scenario of Root Mean Square Prediction Error (RMSPE) and Mean Prediction Error (MPE); shaded regions indicate performance tiers; green for optimal (RMSPE ≤ 41 % or |MPE| ≤ 15 %), yellow for acceptable (41 % < RMSPE ≤ 51 % or 15 % < |MPE| ≤ 30 %), and red for unacceptable (RMSPE > 51 % or |MPE| > 30 %). Error bars show the 95 % confidence intervals for MPE.

**Figure S3.2.** Proportion of virtual individuals (n_tot_ = 1000) receiving 400 mg twice daily for which the predicted (C_min, Pred_) and true (C_min, True_) trough concentrations (i.e., 24 h post dose on day 11 - 264h) fell within the same range (<1100, 1100–2000, >2000 ng/mL; concordant results in green shades, discordant in yellow to red shades), based on therapeutic range in (13). The dotted vertical line represents the separation between 2 dose intakes within the day.
